# Chilblain Lupus Erythematosus

**DOI:** 10.31662/jmaj.2021-0064

**Published:** 2021-09-01

**Authors:** Yoshinosuke Shimamura, Yayoi Ogawa, Hideki Takizawa

**Affiliations:** 1 Department of Nephrology, Teine Keijinkai Medical Center, Sapporo, Japan; 2 Hokkaido Renal Pathology Center, Sapporo, Japan

**Keywords:** Chilblain lupus erythematosus, systemic lupus erythematosus, proteinuria, hematuria

A 41-year-old man presented with a painful rash on his fingers for the last 6 months. He had a history of digital amputation. Notable findings included papuloerythematous plaques on the back of his palms and fingers (Figure 1). Urinalysis revealed proteinuria and microhematuria. The patient was positive for antinuclear (speckled pattern), anti-double-stranded DNA, and anti-Smith antibodies and negative for cryoglobulin and antiphospholipid antibodies. Renal biopsy (Figures 2 and 3) confirmed the diagnosis of systemic lupus erythematosus (SLE) presenting with chilblain lupus erythematosus (CLE) and lupus nephritis class III (A/C) and V as per the Renal Pathology Society/International Society of Nephrology classification. Six months after the initiation of prednisolone and mycophenolate mofetil, proteinuria and hematuria disappeared and the skin rash improved.

CLE is one of the forms of skin manifestations of SLE ^(1)^, is characterized by painful papuloerythematous plaques over the fingers ^(2), (3)^, and can be mistakenly diagnosed as chilblain ^(3)^. Physicians should be aware of CLE because it can be the initial manifestation of SLE and may require further investigation to determine other organ involvement in SLE ^(2), (3)^.

**Figure 1.**
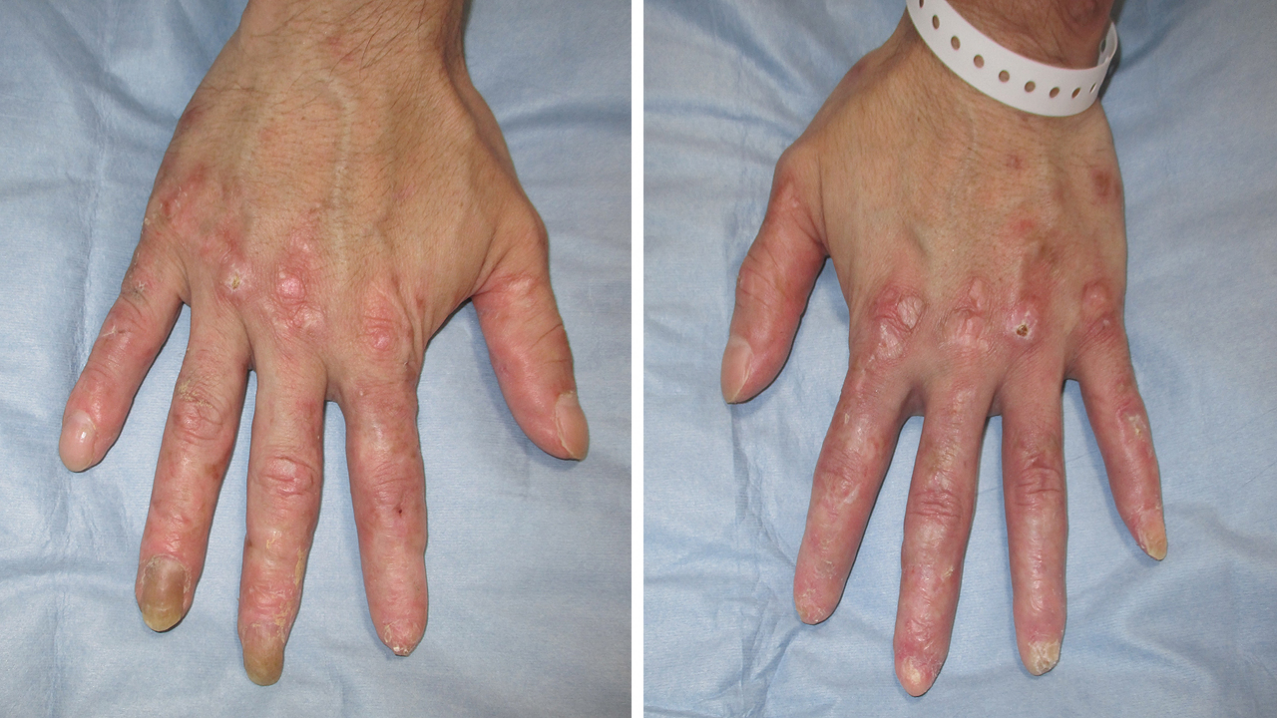
Physical examination showed papuloerythmatous plaques on the backs of the patient’s bilateral fingers.

**Figure 2.**
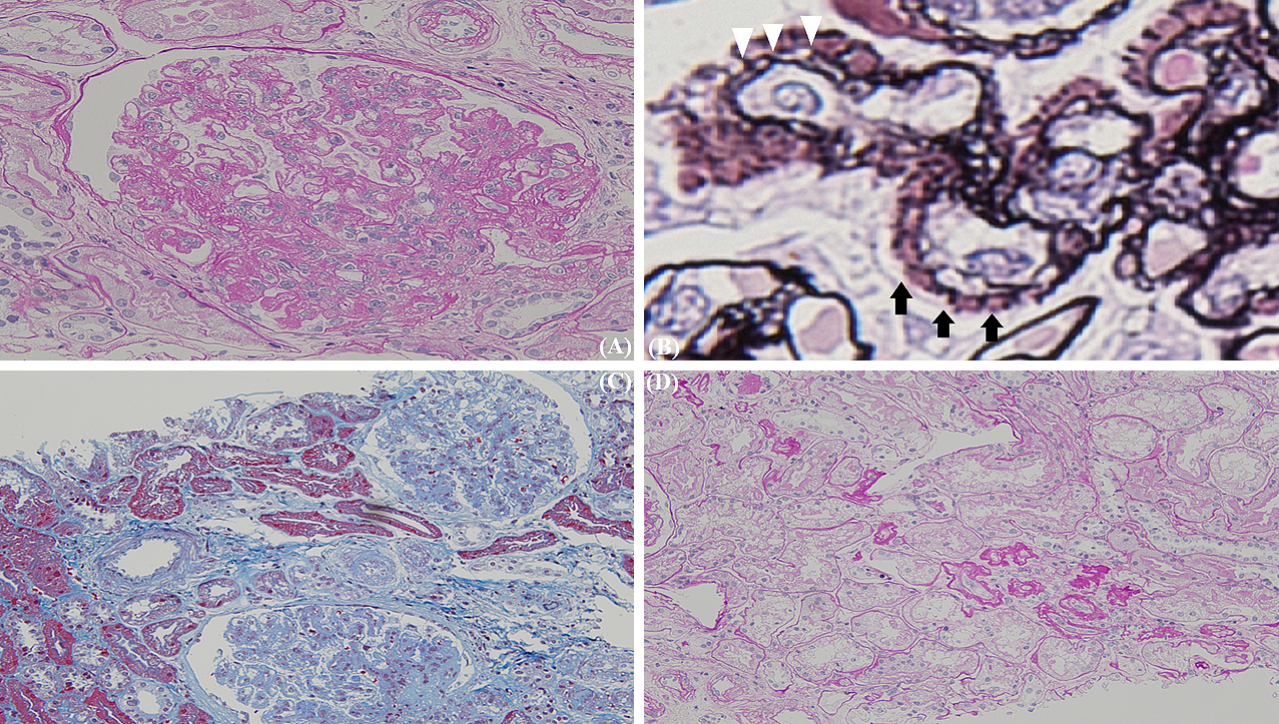
Light microscopy of the kidney showing diffuse mesangial hypercellularity (A, periodic acid-Schiff staining, 400×); spike formation (*black arrows*) and double contouring (*white arrowheads*) of the glomerular basement membrane (B, periodic acid-methenamine-silver staining, 800×); no arterial lesions (C, Masson’s trichrome staining, 400×); mild tubular atrophy (D, periodic acid-Schiff staining, 400×).

**Figure 3.**
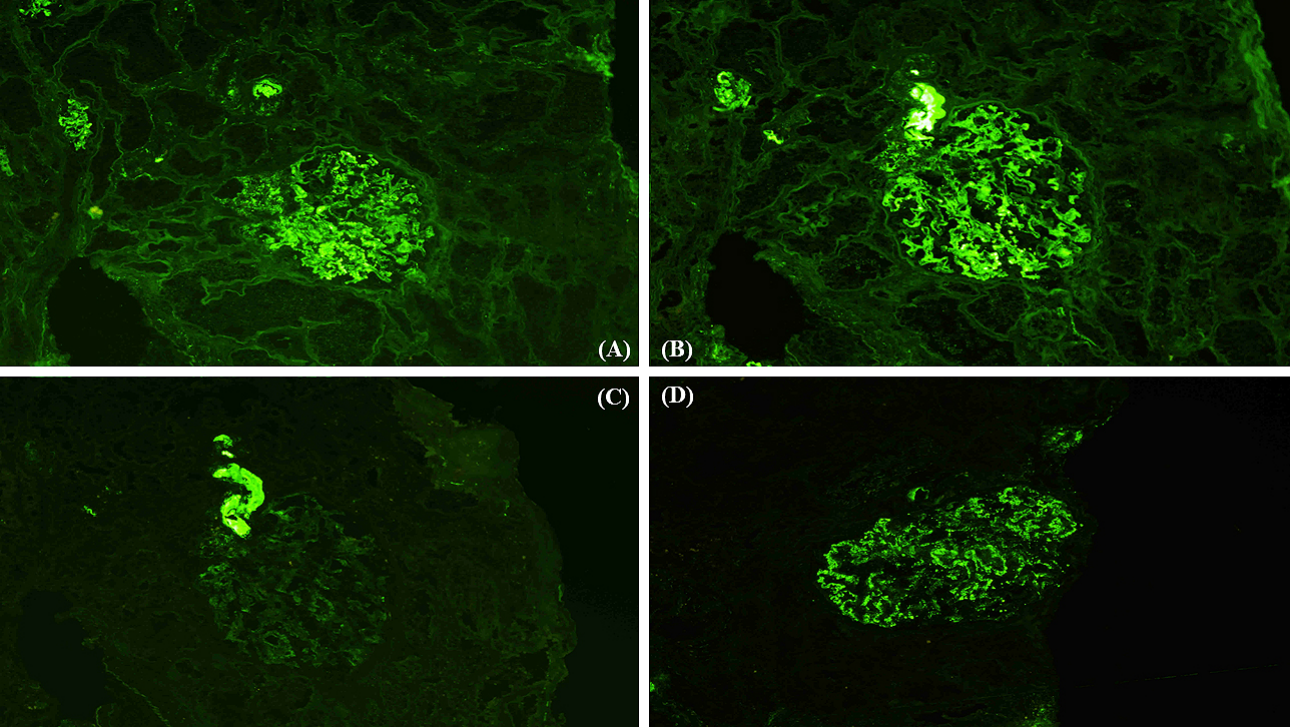
Immunofluorescence microscopy of the kidney showing the depositions of immunoglobulins G, A, and M, and C1q. (A) immunoglobulin G; (B) immunoglobulin A; (C) immunoglobulin M; (D) C1q, 400×.

## Article Information

### Conflicts of Interest

None

### Acknowledgement

We would like to thank Editage (www.editag.jp) for English language editing.

### Author Contributions

Conceptualization YS, HT; Investigation YS, HT; Project administration HT; Resources YO; Supervision HT; Visualization YO; Writing - original draft YS, YO, HT; Writing - review & editing YS, YO, HT.

### Approval by Institutional Review Board (IRB)

2-020083-00 issued by the institutional review board at the Teine Keijinkai Medical Center

### Informed Consent

Written informed consent was obtained from all patients to publish the information, including their photographs.
